# Nomogram prediction of individual prognosis of patients with hepatocellular carcinoma

**DOI:** 10.1186/s12885-017-3062-6

**Published:** 2017-01-31

**Authors:** Gang Wan, Fangyuan Gao, Jialiang Chen, Yuxin Li, Mingfan Geng, Le Sun, Yao Liu, Huimin Liu, Xue Yang, Rui Wang, Ying Feng, Xianbo Wang

**Affiliations:** 1 Statistics Room, Beijing Ditan Hospital, Capital Medical University, No. 8 Jing Shun East Street, 100015 Beijing, China; 2 Center of Integrative Medicine, Beijing Ditan Hospital, Capital Medical University, No. 8 Jing Shun East Street, 100015 Beijing, China; 3 Department of Gastroenterology, Dongzhimen Hospital, Beijing University of Chinese Medicine, No. 5 Hai Yun Cang, 100700 Beijing, China

**Keywords:** Hepatocellular carcinoma, Nomogram, Overall survival

## Abstract

**Background:**

The purpose of this study was to develop an effective nomogram capable of estimating the individual survival outcomes of patients with hepatocellular carcinoma (HCC), and compare the predictive accuracy and discriminative ability with other staging systems.

**Methods:**

The nomogram was established based on a retrospective study of 661 patients newly diagnosed with HCC at the Beijing Ditan Hospital (Beijing, China), Capital Medical University, between October 2008 and July 2012. The predictive accuracy and discriminative ability of the previously developed nomogram were assessed by C-index and calibration curves, and were compared to seven current commonly used staging systems. The results were validated, using a bootstrap approach to correct for bias, in a prospective study of 220 patients consecutively enrolled between August 2012 and March 2013.

**Results:**

Multivariate analysis of the primary cohort for survival analysis identified the independent factors to be aspartate aminotransferase, ɣ-glutamyl transpeptidase, white blood cell count, neutrophil-to-lymphocyte ratio, prothrombin activity, α-fetoprotein, tumor number and size, lymph node metastasis, and portal vein involvement, which were all included to build the nomogram. The calibration curve for predicting the probability of survival showed consistency between the nomogram and the actual observation. The C-index of the nomogram was 0.81 (95% confidence interval, 0.79–0.82), which was statistically better than that of the Tumor, Node, Metastasis staging (0.71), Barcelona Clinic Liver Cancer staging (0.77), Okuda (0.62), Japan Integrated Staging (0.73), Cancer of the Liver Italian Program score (0.76), Chinese University Prognostic Index (0.68), and the Groupe d’ Etude et de Traitement du Carcinome Hepatocellulaire Prognostic classification (0.65) (*p* < 0.001 for all). The results were validated in the prospective validation cohort.

**Conclusions:**

The prognostic nomogram resulted in more accurate individualized risk estimates for overall survival in HCC patients.

## Background

Hepatocellular carcinoma (HCC) is the fifth most common malignancy and the second highest mortality rate among cancers worldwide, accounting for more than 0.5 million deaths annually [1]. Furthermore, the incidence of HCC has been increasing in the last decades [2, 3]. Therefore, HCC has been a major health problem worldwide. In the past decades, several effective therapies have been developed, including surgical resection, liver transplantation, radiofrequency ablation (RFA), microwave ablation, percutaneous ethanol injection (PEI), and transcatheter arterial embolization or chemoembolization (TAE/TACE) [4]. Therefore, it is imperative to determine whether a patient would benefit from aggressive therapies, while avoiding overtreatment. Cancer staging is important for guiding therapeutic interventions and assessing prognosis that could be of significance for both the patients and clinicians in decision-making.

Currently, several staging systems are being used to predict survival in HCC patients, including the Tumor, Node, Metastasis (TNM) staging [5], Barcelona Clinic Liver Cancer (BCLC) staging [6], Okuda [7], Cancer of the Liver Italian Program (CLIP) score [8], Japan Integrated Staging Score (JIS) [9], Chinese University Prognostic Index (CUPI) [10], and the Groupe d’ Etude et de Traitement du Carcinome Hepatocellulaire Prognostic classification (GETCH) [11], all of which have their advantages and disadvantages. The Okuda, CUPI, and GETCH classifications properly stratified the prognosis of patients with advanced or terminal stage [12]. The TNM staging only accounts for tumor-related indicators reflecting the tumor morphology and pathology, without taking the liver functional features into consideration [13]. Meanwhile, these staging systems only serve to stratify patients into various groups with variable outcomes, but could not estimate the individual survival outcomes of HCC.

Nomograms are graphic calculating scales of predictive statistical models to optimize predictive accuracy of individuals [14, 15], and they have been developed for several carcinomas [16–19]. Because nomograms has been demonstrated to provide more precise prediction over the traditional staging systems in many types of cancers, it has been proposed as an alternative method or even as a new standard to guide the administration of appropriate treatment to cancer patients [16, 19, 20]. However, nomograms that predict overall survival (OS) in HCC patients are rare. Although Li shu *et al.* proposed a prognostic nomogram specifically developed for patients with unresectable HCCs after TACE, it did not cover the entire clinical spectrum of HCCs [21]. Patients who were suitable candidates for surgical resection or had advanced/end-stage cancers were excluded. In this study, the specific aim of this analysis was to develop a simple and clinically useful nomogram for patients with HCC and compare the performance of this model with the currently available staging systems.

## Methods

### Patients and design

We retrospectively analyzed 661 patients between October 2008 and July 2012 and prospectively studied 220 patients between August 2012 and March 2013, who were newly diagnosed with HCC at the Beijing Ditan Hospital (Beijing, China), Capital Medical University. The diagnosis of HCC was based on the European Association for the Study of the Liver (EASL) criteria [22]: a histopathologic confirmation, a positive lesion detected by at least 2 different imaging techniques, or a positive lesion detected by 1 imaging technique combined with α-fetoprotein (AFP) >400 ng/ml. The imaging techniques included transabdominal ultrasonography, angiogram, computed tomography (CT) and magnetic resonance imaging (MRI). Patient records and information was anonymized prior to analysis. This project was approved by the ethics committee of the Beijing Ditan Hospital (Beijing, China).

The inclusion criteria were age 18–75 years; newly diagnosed with HCC; and no history of previous anticancer therapy. The exclusion criteria were the diagnosis or history of other malignancies; tumors of uncertain origin or probable metastatic liver tumors; patients with missing key data concerning clinical information and laboratory data; or patients with no follow-up data.

Resection and liver transplantation should be the first option for patients who have the optimal profile. Locoregional approaches including ablation and TAE were used for patients who were not suitable candidates for curative therapies. RFA, PEI, or microwave ablation was performed in HCC patients with 2–3 nodules ≤3 cm. TACE/Lp-TAE were performed in patients with 4 nodules >3 cm, or Child-Pugh A or B. Sorafenib and FOLFOX regimens were considered first-line treatment in patients with distant metastases who can no longer be treated with potentially more effective therapies. End stage includes those patients with severe impairment of liver function (Child-Pugh C) merely received the best supportive care [23, 24].

### Data collection

A standardized data collection form was designed to retrieve all the relevant information on demographic data (age, sex, history of smoking, history of alcohol consumption, family history of HCC, and household registry); laboratory data (alanine aminotransferase [ALT], aspartate aminotransferase [AST], total bilirubin [TBil], serum albumin [ALB], alkaline phosphatase [ALP], ɣ-glutamyl transpeptidase [GGT], prothrombin activity [PTA], international normalized ratio [INR], AFP, white blood cell [WBC] count, absolute neutrophil count [NC], absolute lymphocyte count [LC], absolute platelet count [PLT], neutrophil-to-lymphocyte ratio [NLR]); and tumor-related indicators (tumor size and number, lymph node metastasis, distant metastasis, portal vein involvement). The relevant data were collected from the patient medical records or the hospital database at the time of HCC diagnosis and during the follow-up period. In addition, seven scoring systems associated with clinical prognosis were used at baseline, which were the TNM, BCLC, Okuda, CLIP, JIS, CUPI, and GETCH staging scores, as previously described [5–11].

### Follow-up

All patients were followed-up at least once every 3 months during the first 2 years after treatment, and every 4–6 months annually thereafter. At each of these follow-up visits, a detailed history was taken and a complete physical examination was carried out. Abdominal CT or MRI was also done annually or earlier when tumour recurrence/metastasis was suspected. OS was defined as the interval between diagnosis and death from any cause or until the last known follow-up, obtained from the patient medical records, or through direct contact with the patients or their families.

### Statistical analysis

All the statistical analyses were conducted with SPSS 20.0 statistical package (IBM, Armonk, NY, USA). Continuous variables were presented as mean ± standard deviation or medians with interquartile ranges, while categorical variables as the frequencies or percentages of events. The Student’s *t*-test or Mann–Whitney U test was used for continuous data. The Pearson chi-square or Fisher’s exact tests were used to compare differences in proportion between the groups, as appropriate. Cox univariate and multivariate regression analyses were performed to identify independent risk factors for predicting mortality.

Nomograms were formulated based on the results of the multivariate Cox regression analyses performed using the RMS packages [25] in R version 3.0.2 (http://www.r-project.org/). Final selection of the nomogram model was based on a backward step-down process with the Akaike information criterion [26]. The performance of the nomograms and other seven staging systems for predicting survival were evaluated by the concordance index (C-index), an equivalent variable of the area under curve (AUC) of the receiver operating characteristic (ROC) curve for censored data. The maximum C-index value is 1.0, which indicates a perfect prediction model whereas 0.5 indicates a random chance to correctly predict outcome by the model. Bootstraps with 1,000 resamples were used for validation to correct the C-index and explain the variance due to over-optimism. Comparisons between nomogram models and the other seven staging systems were performed with the rcorrp.cens function in the Hmisc package [27] in R. Calibration curves of the nomogram for 1-, 2-, and 3-year OS were applied to assess the agreement between the predicted survival and the observed survival. Clinical survival outcomes were assessed by Kaplan–Meier analysis and prognostic groups were compared by log-rank test. When externally validating the nomogram, the total points for each patient were computed according to the established nomogram, which were used as factors in the Cox regression model, and the C-index and calibration curves were derived based on the regression analysis. All statistical tests were two-sided with a statistical significance level set at *p* values < 0.05.

## Results

### Patient characteristics and outcomes

In total, 1221 patients newly diagnosed with HCC during the study period were enrolled in the study. Following the exclusion of those who did not meet the inclusion criteria, 661 patients were finally included in the primary cohort, and 220 in the prospective validation cohort. The baseline characteristics of the primary and validation cohorts are listed in Table 1. 356 (40.4%) of the patients had survived, whereas 525 (59.6%) of the patients had died by the end of the 3-year follow-up. The median OS periods were 25.0 months and 21.0 months for the primary and validation cohorts, respectively. The 1-, 2-, and 3-year OS rates were 66.1, 50.8, and 41.6% in the primary cohort, and 63.6, 42.3, and 36.4% in the prospective validation cohort, respectively.

**Table 1:** Patient demographics and clinical characteristics

Patient’s Characteristics	Total	Primary cohort	Prospective validation cohort	*p* value
	(*n* = 881)	(*n* = 661)	(*n* = 220)	
Patient background				
Age, yr	54.5 ± 10.0	54.4 ± 10.0	54.7 ± 9.9	0.746
Gender (Male/Female)	737/144 (83.7%/16.3%)	551/110 (83.4%/16.6%)	186/34 (84.6%/15.4%)	0.680
Family history of HCC (Yes/No)	115/766 (13.0%/87.0%)	85/576 (12.9%/87.1%)	30/190 (13.6%/86.4%)	0.767
History of smoking (Yes/No)	342/539 (38.8%/61.2%)	254/407 (38.4%/61.6)	88/132 (40.0%/60.0%)	0.678
History of alcohol use (Yes/No)	344/537 (39.0%/61.0%)	262/399 (39.6%/60.4%)	82/138 (37.3%/62.7%)	0.534
Cirrhosis (Yes/No)	720/161 (81.7%/18.3%)	536/125 (81.1%/18.9%)	184/36 (83.6%/16.4%)	0.397
Cause of HCC				
Hepatitis B (Yes/No)	780/101 (88.5%/11.5%)	582/79 (88.0%/12.0%)	198/22 (90.0%/10.0%)	0.431
Hepatitis C (Yes/No)	69/812 (7.8%/92.2%)	52/609 (7.9%/92.1%)	17/203 (7.7%/92.3%)	0.947
Alcohol Liver (Yes/No)	121/760 (13.7%/86.3%)	94/567 (14.2%/85.8%)	27/193 (12.3%/87.7%)	0.467
other cause (Yes/No)	3/878 (0.3%/99.7%)	3/658 (0.4%/99.6%)	0/220 (0.0%/100.0%)	0.578
Laboratory data				
ALT, IU/L	36.5 (24.8,59.4)	36.4 (24.8,60.1)	36.5 (24.7,57.6)	0.972
AST, IU/L	45.3 (29.8,74.2)	45.6 (29.4,75.7)	42.7 (30.0,69.9)	0.450
TBIL, μmol/L	20.2 (13.5,31.1)	21.0 (14.2,33.4)	17.3 (12.5,25.8)	<0.001
ALB, g/L	36.6 ± 6.9	36.4 ± 7.2	37.1 ± 6.2	0.165
ALP, IU/L	123.9 ± 82.0	124.9 ± 82.3	121.0 ± 81.1	0.552
GGT, IU/L	64.3 (30.8,138.3)	60.6 (29.9,133.7)	71.5 (37.1,143.9)	0.042
WBC, 10^9^/L	4.7 ± 1.9	4.6 ± 1.9	4.8 ± 2.0	0.269
NC, 10^9^/L	2.9 ± 1.5	2.9 ± 1.5	3.0 ± 1.5	0.554
LC, 10^9^/L	1.23 ± 0.61	1.20 ± 0.61	1.30 ± 0.59	0.042
PLT, 10^9^/L	112.3 ± 67.9	109.2 ± 64.5	121.5 ± 76.8	0.034
NLR	2.9 ± 2.0	2.9 ± 2.0	2.7 ± 1.8	0.255
Cr, μmoI/L	70.5 ± 31.9	71.7 ± 35.2	66.8 ± 18.3	0.008
PTA, %	75.7 ± 18.6	74.8 ± 18.8	78.4 ± 17.4	0.012
INR	1.2 ± 1.6	1.2 ± 1.8	1.2 ± 0.3	0.453
AFP, ng/mL (<400/ ≥ 400)	221/660 (25.1%/74.9%)	161/500 (24.4%/75.6%)	60/160 (27.3%/72.7%)	0.388
Tumor-related indicators				
Tumor number (<3/ ≥ 3)	310/556 (35.8%/64.2%)	220/432 (33.7%/66.3%)	90/124 (42.1%/57.9%)	0.028
Tumor size,cm (<5/ ≥ 5)	262,537 (32.8%/67.2%)	192/415 (31.6%/68.4%)	70/122 (36.5%/63.5%)	0.214
Lymph node metastasis (Yes/No)	79/802 (9.0%/91.0%)	54/607 (8.2%/91.8%)	25/195 (11.4%/88.6%)	0.151
Portal vein involvement (Yes/No)	227/654 (25.8%/74.2%)	164/497 (24.8%/75.2%)	63/157 (28.6%/71.4%)	0.261

Data are presented as n (%), mean ± SD, or median (interquartile range)

*Abbreviations*: *ALT* alanine aminotransferase, *AST* aspartate aminotransferase, *TBil* total bilirubin, *ALB* serum albumin, *ALP* alkaline phosphatase, *GGT* ɣ-glutamyl transpeptidase, *WBC* white blood cell count, *NC* absolute neutrophil count, *LC* absolute lymphocyte count, *PLT* platelet count, *NLR* neutrophil-lymphocyte ratio, *Cr* serum creatinine, *PTA* prothrombin activity, *INR* international normalized ratio, *AFP* alpha fetoprotein

### Univariate and multivariate analyses in the primary cohort

For OS, the significant inferior prognostic factors included the male sex, history of alcohol consumption, ALT, AST, TBil, ALB, ALP, GGT, WBC, NC, LC, NLR, Cr, PTA, AFP ≥ 400 ng/mL, tumor number ≥ 3, tumor diameter ≥ 5 cm, lymph node metastasis, and portal vein involvement (*p* < 0.05). The above variables were entered into multivariate Cox proportional hazard regression analyses. The results indicated AST, GGT, WBC, NLR, PTA, AFP ≥ 400 ng/mL, tumor number ≥ 3, tumor diameter ≥ 5 cm, lymph node metastasis, and portal vein involvement to be independent prognostic variables. The detailed results of the multivariate analysis are shown in Table 2.

**Table 2:** Univariate and multivariate Cox regression analyses for OS in patients with HCC from the primary cohort (*n* = 661)

	Univariate analysis		Multivariate analysis	
	HR (95% CI)	*p* value	HR (95% CI)	*p* value
Factors Selected				
AST, IU/L	1.00 (1.00-1.00)	<0.001	1.01 (1.00-1.01)	0.010
GGT, IU/L	1.00 (1.00-1.00)	<0.001	1.01 (1.00-1.01)	0.001
WBC, 10^9^/L	1.16 (1.10-1.22)	<0.001	1.09 (1.02-1.16)	0.007
NLR	1.33 (1.28-1.38)	<0.001	1.15 (1.10-1.21)	<0.001
PTA, %	0.98 (0.97-0.98)	<0.001	0.98 (0.97-0.99)	<0.001
AFP ≥ 400 ng/mL	2.82 (2.28-3.49)	<0.001	1.36 (1.06-1.76)	0.017
Tumor number ≥ 3	2.19 (1.79-2.69)	<0.001	1.27 (1.01-1.61)	0.044
Tumor size ≥ 5 cm	3.12 (2.52-3.86)	<0.001	1.56 (1.22-1.99)	<0.001
Lymph node metastasis	4.22 (3.12-5.72)	<0.001	1.66 (1.18-2.34)	0.004
Portal vein involvement	9.45 (7.48-11.95)	<0.001	4.85 (3.62-6.49)	<0.001
Factors not Selected				
Age, yr	0.99 (0.98-1.00)	0.075		
Male	0.75 (0.57-1.00)	0.047		
Familyhistory of HCC	1.16 (0.87-1.55)	0.310		
History of smoking	1.17 (0.96-1.44)	0.127		
History of alcohol use	1.46 (1.19-1.79)	<0.001		
ALT, IU/L	1.00 (1.00-1.00)	0.033		
TBIL, μmol/L	1.01 (1.01-1.01)	<0.001		
ALB, g/L	0.96 (0.95-0.98)	<0.001		
ALP, IU/L	1.00 (1.00-1.01)	<0.001		
NC, 10^9^/L	1.36 (1.28-1.45)	<0.001		
LC, 10^9^/L	0.55 (0.46-0.67)	<0.001		
PLT, 10^9^/L	1.00 (1.00-1.00)	0.060		
Cr, μmoI/L	1.00 (1.00-1.01)	<0.001		
INR	1.01 (0.96-1.05)	0.795		

*Abbreviations*: *ALT* alanine aminotransferase, *AST* aspartate aminotransferase, *TBil* total bilirubin, *ALB* serum albumin, *ALP* alkaline phosphatase, *GGT* ɣ-glutamyl transpeptidase, *WBC* white blood cell count, *NC* absolute neutrophil count, *LC* absolute lymphocyte count, *PLT* platelet count, *NLR* neutrophil-lymphocyte ratio, *Cr* serum creatinine, *PTA* prothrombin activity, *INR* international normalized ratio, *AFP* alpha fetoprotein, *HR* hazard ratio, *CI* confidence interval

### Prognostic nomogram for survival

**Fig. 1:**
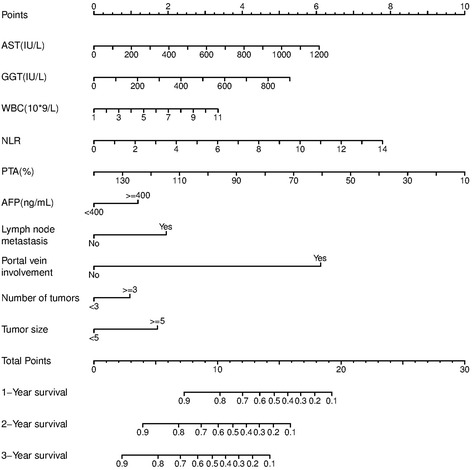
A hepatocellular carcinoma survival nomogram is depicted. To use the nomogram, the value of an individual patient is located on each variable axis, and a line is drawn upward to determine the number of points received for the value of each variable. The sum of these numbers is located on the total point axis, and a line is drawn downward to the survival axes to determine the likelihood of 1-, 2-, and 3-year survivals. AST, aspartate aminotransferase; GGT, γ-glutamyl transpeptidase; WBC, white blood cell; NLR, neutrophil-to-lymphocyte ratio; PTA, prothrombin activity; AFP, alpha fetoprotein

The coefficients obtained from the Cox regression model were used to construct the nomograms for OS (Fig. 1). Each subtype within the variables was assigned a score. By adding up the total score from all the variables and locating it to the total point scale, we could determine the probabilities of the outcomes by drawing a vertical line to the total score. The nomograms included three liver function indices (AST, GGT, PTA), two inflammatory indices (WBC, NLR), and five tumor-related indicators (AFP, tumor number, tumor size, lymph node metastasis, and portal vein involvement), of which PTA, NLR, and portal vein involvement were the most important contributing factors for OS prediction. Details concerning the point assignment from the nomograms and the prognostic score are shown in Table 3.

**Table 3:** Point assignment from nomograms and prognostic scores

Liver function index						Inflammatory index				Tumor index	Points
AST	Points	GGT	Points	PTA	Points	WBC	Points	NLR	Points		
0	0	0	0	10	10	1	0	0	0	AFP	
100	1	100	1	20	9	2	0	1	1	<400	0
200	1	200	1	30	8	3	1	2	1	≥400	1
300	2	300	2	40	8	4	1	3	2	Lymph node metastasis	
400	2	400	2	50	7	5	1	4	2	No	0
500	3	500	3	60	6	6	2	5	3	Yes	2
600	3	600	4	70	5	7	2	6	3	Portal vein involvement	
700	4	700	4	80	5	8	2	7	4	No	0
800	4	800	5	90	4	9	3	8	4	Yes	6
900	5	900	5	100	3	10	3	9	5	Tumor number	
1000	5			110	2	11	3	10	6	<3	0
1100	6			120	2			11	6	≥3	1
1200	6			130	1			12	7	Tumor size	
				140	0			13	7	<5	0
								14	8	≥5	2

*Abbreviations*: *AST* aspartate aminotransferase, *GGT* ɣ-glutamyl transpeptidase, *WBC* white blood cell count, *NLR* neutrophil-lymphocyte ratio, *PTA* prothrombin activity, *AFP* alpha fetoprotein

### Validation of the prognostic nomogram

**Fig. 2:**
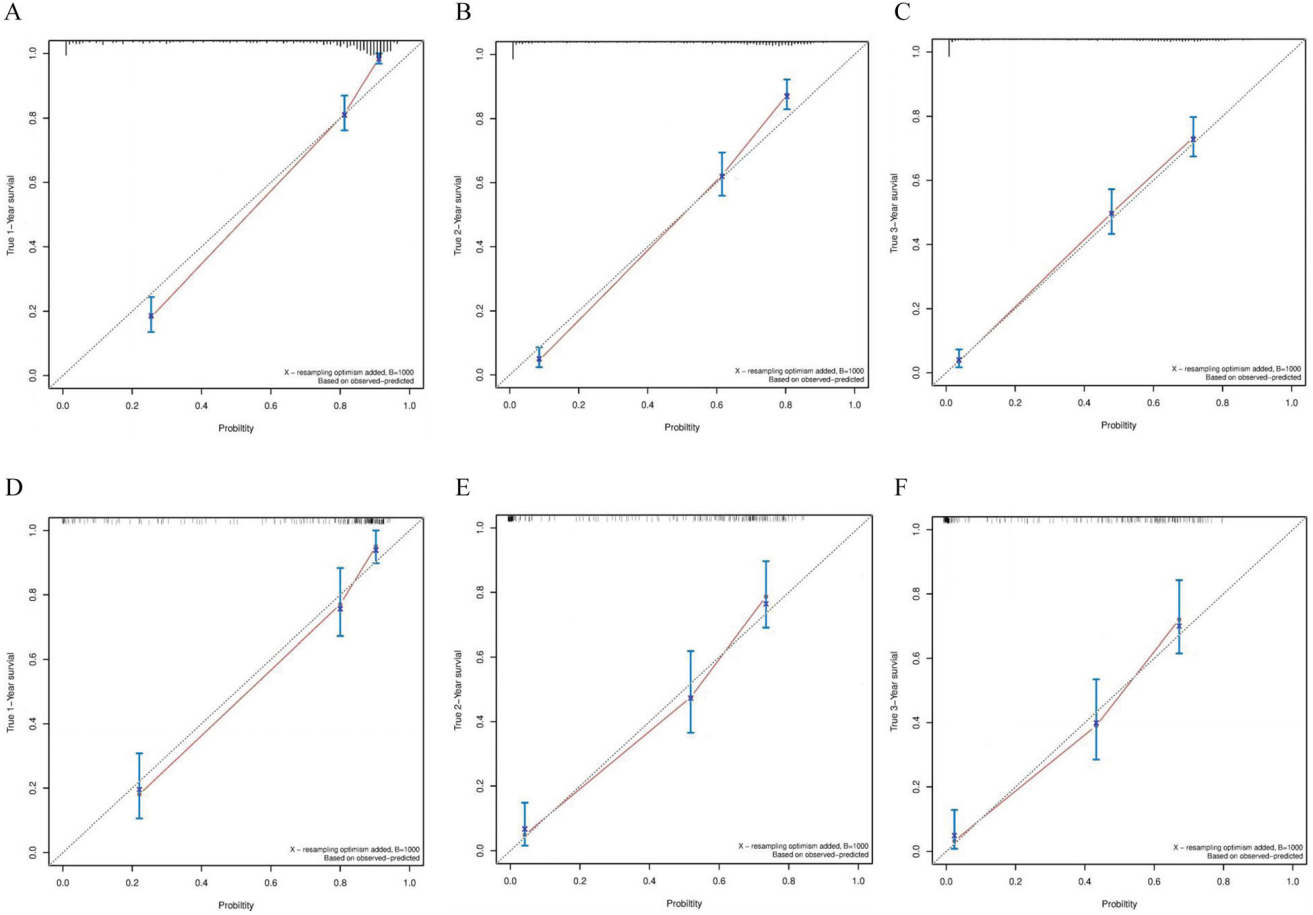
The calibration curve of overall survival at 1, 2, and 3 years for the primary cohort (**a**-**c**) and the prospective validation cohort (**d**-**f**). Nomogram-predicted probability of survival is plotted on the x-axis, and the actual survival is plotted on the y-axis. Dashed lines along the 45° line through the point of origin represent the perfect calibration models where the predicted probabilities are identical to the actual probabilities

The C-index for the established nomogram for predicting the OS was 0.81 (95% confidence interval (CI), 0.79–0.82) in the primary cohort. When the validation cohort was subjected to the nomogram, the C-index was 0.78 (95% CI, 0.74–0.82), which was greater than 0.7, suggesting the suitability of the new model for patients with HCC. The calibration plots showed fair agreements between the nomogram predictions and actual observations for the 1-, 2-, and 3-year OS in the primary cohort (Fig. 2a-c) and the prospective validation cohort (Fig. 2d-f).

**Fig. 3:**
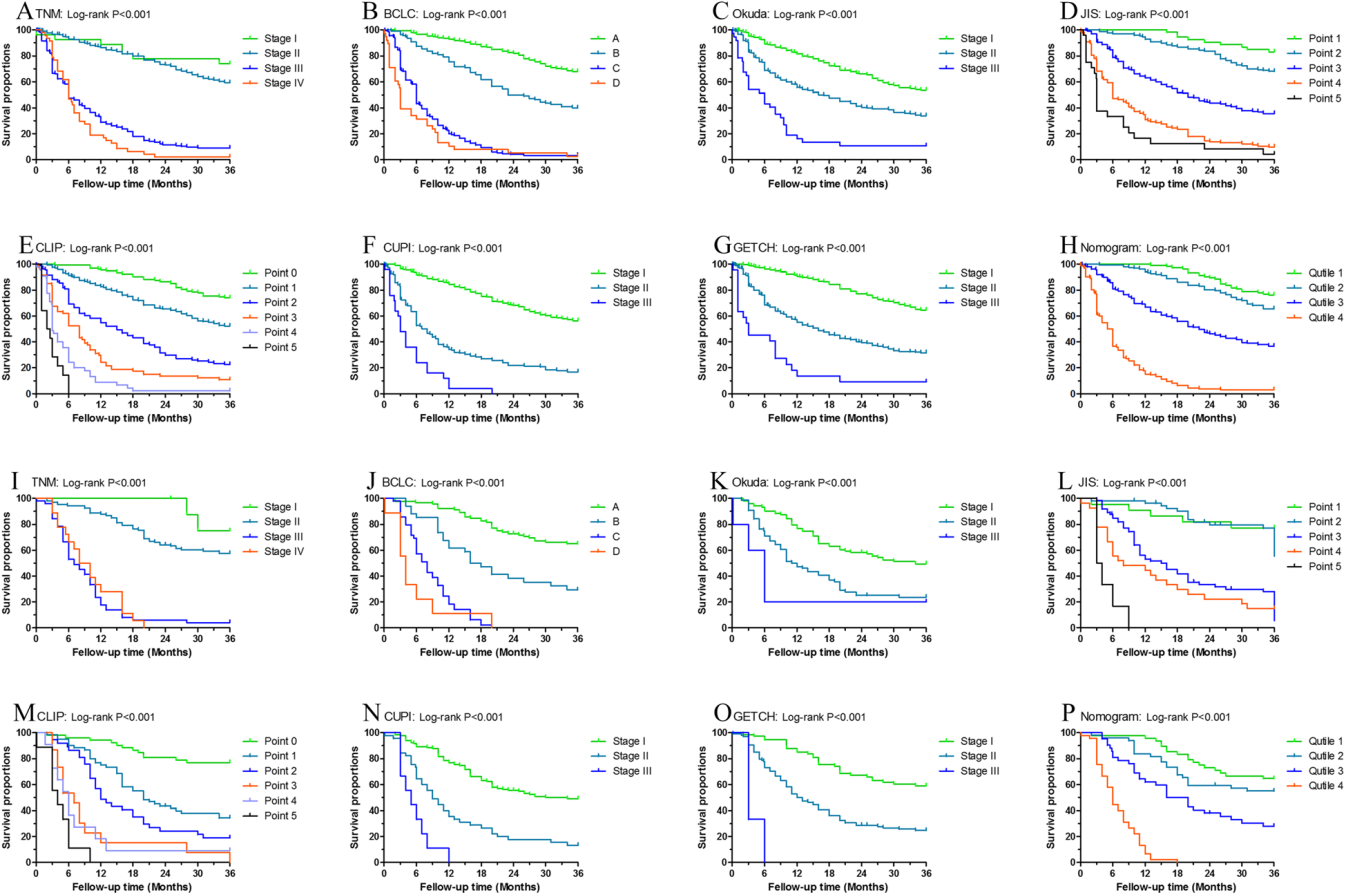
Kaplan–Meier curves of risk group stratification for OS in the primary cohort (**a**-**h**) and the prospective validation cohort (**i**-**p**) categorized according to different staging systems. **a**,**i** Tumor, Node, Metastasis (TNM); **b**,**j** Barcelona Clinic Liver Cancer (BCLC); **c**,**k** Okuda; **d**,**l** Japan Integrated Staging Score (JIS); **e**,**m** Cancer of the Liver Italian Program (CLIP); **f**,**n** Chinese University Prognostic Index (CUPI); **g**,**o** Groupe d’ Etude et de Traitement du Carcinome Hepatocellulaire Prognostic classification (GETCH); and **h**,**p** the prognostic nomogram

According to the total scores, patients were divided into four quartiles (quartile 1: 0–7; quartile 2: 8–10; quartile 3: 10–15; and quartile 4: > 15). After dividing the survival rates into quartiles, we further identified the prognostic discrimination of the nomograms by Kaplan–Meier analysis. The nomogram could accurately stratify patients into the 4 risk groups with significant differences in the 1-, 2-, and 3-year OS rates in the primary (3-year OS rate: 76.2% in quartile 1, 65.6% in quartile 2, 25.7% in quartile 3, and 3.2% in quartile 4; *p* < 0.001) and the validation cohort (3-year OS rate: 64.6% in quartile 1, 55.1% in quartile 2, 28.6% in quartile 3, and 0% in quartile 4; *p* < 0.001) (Fig. 3h,p).

### The performance of the nomogram compared to other staging systems

As shown in Fig. 3a-p, the Kaplan–Meier curves were generated for all the staging systems in the primary and validation cohorts. Although all the staging systems showed good prognostic stratification (*p* < 0.001 for all cases) in the primary cohort, some overlapping of the survival curves for TNM, BCLC, JIS, and CLIP was observed. In the validation cohort, all the staging systems also showed clear prognostic stratification (*p* < 0.001); however, some overlapping of the survival curves was observed for the TNM, BCLC, Okuda, JIS, and CLIP. In both cohorts, the TNM staging was not good at stratifying patients with stage III–IV, and the BCLC was not good at stratifying the patients between stages C and D. Moreover, the Okuda, CUPI, and the GETCH classifications could stratify patients with stage I–III to a certain extent, whereas they were unsatisfactory in stratifying patients in the early stages of HCC in both cohorts.

Our nomogram displayed better accuracy in predicting survival of patients with HCC in both cohorts. As shown in Table 4, the nomogram for OS had a bootstrap-corrected C-index of 0.81, which was significantly better than that of the TNM (0.71), BCLC (0.77), Okuda (0.62), JIS (0.62), CLIP (0.76), CUPI (0.68), and GETCH (0.65) staging systems in the primary cohort (*p* < 0.001 for all). In the prospective validation cohort, the nomograms had the highest areas under the curve (0.78), but without statistical significance only in comparison to BCLC.

**Table 4:** The predictive discrimination ability of the nomogram compared to the TNM, BCLC, Okuda, JIS, CLIP, CUPI, and GETCH staging systems in the primary and validation cohorts

	C-index	95% CI for C-index		Goodness of Fit		Comparison of models			
		Lower	Upper	LR	R^2^	Dxy	SD	Z	*p* value
Primary cohort (*n* = 661)									
TNM	0.71	0.69	0.73	264.05	0.358	0.40	0.03	13.28	<0.001
BCLC	0.77	0.75	0.79	368.95	0.462	0.20	0.03	5.87	<0.001
Okuda	0.62	0.60	0.65	70.3	0.111	0.55	0.02	23.51	<0.001
JIS	0.73	0.71	0.76	227.23	0.317	0.34	0.03	11.27	<0.001
CLIP	0.76	0.74	0.78	303.8	0.400	0.28	0.03	9.38	<0.001
CUPI	0.68	0.66	0.70	178.33	0.259	0.45	0.03	17.29	<0.001
GETCH	0.65	0.62	0.67	103.83	0.160	0.52	0.02	21.98	<0.001
Nomogram	0.81	0.79	0.82	580.13	0.525	-	-	-	-
Prospective validation cohort (*n* = 220)									
TNM	0.74	0.71	0.77	107.94	0.447	0.12	0.06	2.14	0.035
BCLC	0.77	0.73	0.81	124.83	0.496	0.06	0.05	1.24	0.220
Okuda	0.62	0.57	0.67	20.32	0.105	0.49	0.04	12.07	<0.001
JIS	0.71	0.67	0.76	69.15	0.315	0.31	0.05	6.48	<0.001
CLIP	0.75	0.71	0.80	95.42	0.407	0.13	0.05	2.49	0.014
CUPI	0.64	0.60	0.69	44.17	0.215	0.44	0.04	9.82	<0.001
GETCH	0.65	0.60	0.69	35.99	0.179	0.43	0.05	9.23	<0.001
Nomogram	0.78	0.74	0.82	119.91	0.482	-	-	-	-

## Discussion

In this study, we established a novel, easy-to-use, and effective nomogram capable of estimating individual survival outcomes for HCC. Moreover, a robust HCC nomogram including the inflammatory indices (WBC, NLR) was developed to improve the predictive power of the current prognostic scores.

Distinct from other solid cancers, the prognosis for HCC patients relies not only on tumor progression but also on the extent of liver dysfunction; approximately 70 to 90% of HCCs occur in the context of chronic liver inflammation and cirrhosis [28, 29]. Consequently, staging systems such as TNM that depend solely on pathological characteristics retain limited prognostic impact on HCC [13]. A number of alternative systems have been proposed for HCC, including the BCLC, CLIP, CUPI, and JIS. However, there is no universally accepted consensus about the best staging system for predicting the outcome of HCC patients.

Numerous clinical and experimental data demonstrated that host inflammatory response to cancer cells is associated with tumor progression [30, 31]. The link between inflammation and cancer is well established. Various markers of systemic inflammation response, including WBC count [32, 33], cytokines [34, 35], and absolute count of blood neutrophils or lymphocytes as well as the neutrophil-to-lymphocyte (NLR) ratio [36–38] have been explored for their prognostic impact in various cancer populations including HCC. In this study, we also found that the WBC count and NLR have moderate contributions to the nomogram prediction of OS. Elevated neutrophils are regarded as a reservoir of the circulating vascular endothelial growth factor, which plays a key role in the promotion of angiogenesis [39], and neutrophils could contribute to metastasis by promoting the motility of tumor cells and the adhesion of metastatic tumor cells to liver sinusoids [40, 41]. Conversely, reduced lymphocyte infiltration, reflecting the suppression of the host immune surveillance, has been shown to attenuate lymphocyte-mediated antitumor immune response [42]. The presence of high intratumoral activated CD8 cytotoxic cells is associated with improved survival in HCC patients [43]. Consequently, when taken together, NLR could reflect the balance between host inflammation and immunity, which has been reported to be a predictor of survival in HCC patients who underwent hepatic resection, RFA, TACE, and liver transplantation [36, 44–46]. In the future, manipulating the inflammatory status and the immune function of HCC patients might be a promising strategy for further improving the clinical outcomes.

The proposed nomogram included three liver function indices (AST, GGT, PTA), five tumor-related indicators (AFP, tumor number and size, lymph node metastasis, and portal vein involvement), and two inflammatory indices (WBC, NLR), which performed well in predicting the survival outcome of HCC patients, and the prediction was supported by the C-index (0.82 and 0.78 for the primary and validation cohorts, respectively) and the calibration curves. In the current study, the nomogram showed the highest predictive accuracy for OS in patients with HCC, compared to the other seven staging systems. Although there was no statistical significance in comparison to the BCLC in the validation cohort, it is worth noting that the nomogram could more effectively stratify patients with advanced stage cancers compared to the TNM, BCLC, Okuda, and CLIP, and more effectively stratify patients in the early stages of HCC than the Okuda, CUPI, and GETCH in both cohorts.

Our nomogram has some limitations. First, the nomogram was established based on a single-center cohort study. Second, the nomograms only included basic clinical and laboratory data. However, the present study aimed to build reliable prediction models. Objective variables are therefore the ideal factors to be included in the models, while subjective variables might negatively affect the models due to inevitable bias. Third, the study was conducted retrospectively and selection bias might exist. However, we have included a relatively large training cohort to build the nomograms and validated them by a prospective dataset. The results consistently showed the satisfactory performance of the established models.

## Conclusions

In conclusion, we developed and validated nomograms predicting individual prognosis in patients with HCC. The proposed nomogram in this study provided better predictive accuracy and discrimination than the TNM, BCLC, Okuda, JIS, CLIP, CUPI, and GETCH staging systems, and it offers a useful tool for providing patient counseling and timing surveillance, as well as clinical assessments. In order to standardize the use of this nomogram, validation with data from other institutions and other patient groups is required.

## Abbreviations

AFP: Alpha fetoprotein
ALB: Albumin
ALP: Alkaline phosphatase
ALT: Alanine aminotransferase
AST: Aspartate aminotransferase
BCLC: Barcelona Clinic Liver Cancer
CIs: Confidence intervals
CLIP: Cancer of the Liver Italian Program
CUPI: Chinese University Prognostic Index
EASL: The European Association for the Study of the Liver
GETCH: Groupe d’Etude et de Traitement du Carcinome Hépatocellulaire
GGT: ɣ-Glutamyltranspeptidase
HCC: Hepatocellular carcinoma
HRs: Hazard ratios
INR: International normalized ratio
JIS: Japan integrated staging
LC: Absolute lymphocyte count
NC: Absolute neutrophil count
NLR: Neutrophil-to-lymphocyte ratio
OS: Overall survival
PLT: Absolute platelet count
PTA: Prothrombin activity
RFA: Radiofrequency ablation
TACE: Transcatheter arterial chemoembolization
TBil: Total bilirubin
TNM: The Tumor, node, metastasis
WBC: White blood cell
